# Recent Advances in Metal Complexes for Antimicrobial Photodynamic Therapy

**DOI:** 10.1002/cbic.202200796

**Published:** 2023-07-17

**Authors:** Thomas W. Rees, Po‐Yu Ho, Jeannine Hess

**Affiliations:** ^1^ The Francis Crick Institute 1 Midland Road London NW1 1AT UK; ^2^ Department of Chemistry King's College London Britannia House, 7 Trinity Street London SE1 1DB UK

**Keywords:** antibiotics, antimicrobials, bacteria, metal complexes, photodynamic therapy

## Abstract

Antimicrobial resistance (AMR) is a growing global problem with more than 1 million deaths due to AMR infection in 2019 alone. New and innovative therapeutics are required to overcome this challenge. Antimicrobial photodynamic therapy (aPDT) is a rapidly growing area of research poised to provide much needed help in the fight against AMR. aPDT works by administering a photosensitizer (PS) that is activated only when irradiated with light, allowing high spatiotemporal control and selectivity. The PS typically generates reactive oxygen species (ROS), which can damage a variety of key biological targets, potentially circumventing existing resistance mechanisms. Metal complexes are well known to display excellent optoelectronic properties, and recent focus has begun to shift towards their application in tackling microbial infections. Herein, we review the last five years of progress in the emerging field of small‐molecule metal complex PSs for aPDT.

## Graphical abstract

A review of the last five years of progress in the field of metal complexes as photosensitizers (PSs) for antimicrobial photodynamic therapy (aPDT). The strategies used to develop new and potent PSs for aPDT to combat antimicrobial resistance (AMR) are assessed herein.

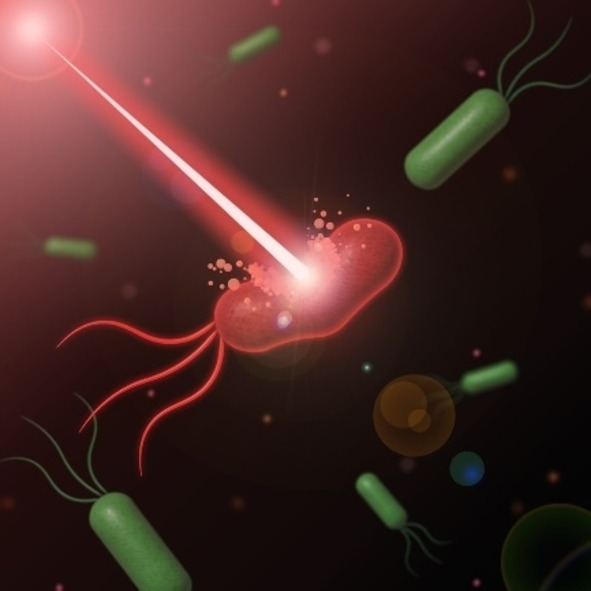

## 1 Introduction

### 1.1 Antimicrobial resistance

The post‐antibiotic era is fast approaching with no sign of an end to the ever‐growing global problem of drug resistant bacteria. In 2019 there were more than 1 million deaths worldwide due to antimicrobial resistance (AMR)^[1]^ and this number is predicted to grow to 10 million by 2050.^[2]^ There have only been two new classes of antibiotics approved for the clinic in the last 30 years.^[3]^ New classes of antibiotics with novel modes of action are therefore desperately needed. Developing new broad‐spectrum antibiotics is however very challenging. There are numerous pathogenic bacterial strains, and although they can be broadly divided by their properties into categories such as Gram‐positive and Gram‐negative, within these categories they can be extremely heterogenous. Finding targets common to, as well as drugs which can effectively treat, all pathogenic bacteria is a huge challenge. In addition, bacteria evolve rapidly presenting an ever‐shifting target. As a result, bacteria have developed various modes of resistance to nearly all types of antibiotics.[^2^, ^4^, ^5^, ^6^] The four main mechanisms of resistance are antibiotic inactivation (*e. g*. β‐lactamases), reduced uptake (*e. g*. efflux pumps), formation of biofilms, and target site modifications (alterations to target proteins etc.).[^4^, ^6^] Developing drugs which can bypass these modes of resistance, is therefore key to the future of antibiotics. Finally, even if we can produce an effective broad‐spectrum antibiotic, the complex involvement of the microbiome in human health means that indiscriminate bactericidal activity could also be very harmful.[^7^, ^8^]

### 1.2 Photodynamic therapy

**Figure 1:**
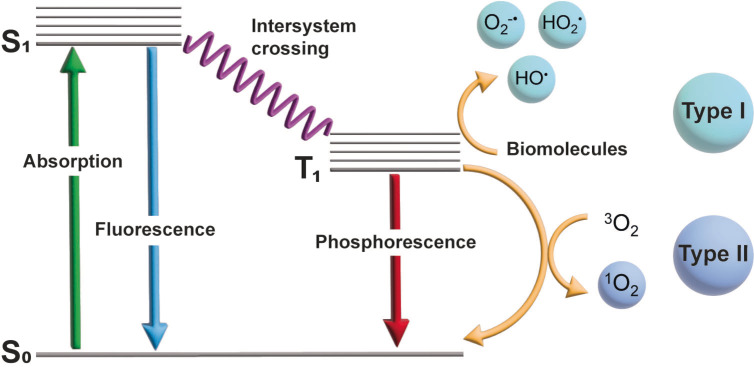
Simplified Jablonski diagram and the process of ROS generation in PDT.

Although the term photodynamic therapy (PDT) was coined in 1904,[^9^, ^10^] it wasn't until 1993 that the first such drug entered clinical use for the treatment of bladder cancer.[^10^, ^11^] PDT is a promising modality now used in the clinic for the treatment of various pathologies.[^11^, ^12^, ^13^, ^14^, ^15^] Recent years have seen an explosion of interest, particularly in the fields of metal complexes and nanomaterials for the PDT of cancer.[^11^, ^16^, ^17^, ^18^, ^19^, ^20^] In PDT, a photosensitizer (PS) is activated by light to produce reactive oxygen species (ROS).[^9^, ^11^] The ROS cause indiscriminate damage to biomolecules, but only at very short distance due to their short lifetime and therefore small diffusion radius (∼3 μs, ∼100 nm for singlet oxygen (^1^O_2_)).[^21^, ^22^] This allows for precise spatiotemporal control, and therefore helps to avoid off‐target toxicity. Figure 1 shows a simplified Jablonski diagram illustrating the process of ROS generation in PDT. The PS is excited to the singlet excited state (S_1_) from the ground state (S_0_) by absorption of a photon. The excited electron then undergoes intersystem crossing to the triplet excited state (T_1_). In Type I PDT the excited electron then reacts with the surrounding biomolecules to generate radical ROS such as superoxide (O_2_^⋅−^), perhydroxyl (HO_2_^⋅^), and hydroxyl (HO^⋅^). Alternatively, in Type II PDT the electron relaxes to the ground state and energy is transferred to a molecule of triplet oxygen (^3^O_2_) to generate highly reactive ^1^O_2_.[^9^, ^11^, ^21^] ROS are known to damage important cell components such as DNA, proteins, and lipids which has been shown to cause cell death in both eukaryotes and prokaryotes.[^23^, ^24^, ^25^]

### 1.3 Photoactive metal complexes

Metal complexes have been used in medicine for at least a century. Indeed, the early antibiotic Salvarsan, developed for the treatment of syphilis is an arsenic complex.[^26^, ^27^] Metal complexes continue to be used in the clinic today. The well‐known metallodrug Pepto‐Bismol (bismuth subsalicylate) is still widely available for indigestion more than 100 years since its first production. Metal complexes are also often employed as contrast agents for techniques such as radioimaging and MRI.^[28]^ One of the most important examples of the past century is cisplatin, an extremely effective platinum‐based cancer treatment, which acts by binding to DNA preventing cell replication.[^28^, ^29^] In the past few decades, metal complexes have become a massive area of research for manifold applications. For example: catalysis, solar energy conversion, and lighting in materials chemistry as well as for various biomedical applications including imaging and therapy.[^28^, ^30^, ^31^, ^32^, ^33^, ^34^, ^35^] Metal complexes are particularly effective for photochemical applications compared to organic compounds due to their readily tunable absorption, emission, and oxidation states as well as their large stokes shifts. Metal complexes also typically allow a modular approach to construction due to the process of decorating a metal center with ligands. This provides extreme diversity in shape and physiochemical properties.[^30^, ^33^] Ruthenium polypyridyl complexes are one of the most explored classes of complex especially due to their photophysical properties and much attention has been paid to their application in biomedicine.[^36^, ^37^, ^38^] In seminal work by the Barton group a complex of ruthenium with a large aromatic ligand was developed, whose emission is enhanced on intercalation with DNA.^[39]^ The group went on to develop rhodium complexes, which target DNA mismatches as possible cancer therapeutics.^[40]^ Another major contribution to the field by the Meggers group investigated the role of the octahedral structure of ruthenium complexes as protein kinase inhibitors.^[41]^ More recently, many groups have developed complexes of various metals for cancer PDT.[^41^, ^42^, ^43^, ^44^] Several reviews and perspectives focusing on this field are referenced here.[^45^, ^46^, ^47^, ^48^] The ruthenium complex TLD‐1433 developed by the McFarland group is particularly noteworthy as it has entered clinical trials for PDT of bladder cancer.^[42]^ This complex was also shown in its first report to be a highly effective aPDT agent against *Staphylococcus aureus*.^[43]^ The field of small molecule metal complexes for aPDT has not developed as significantly as in PDT for cancer. A large focus in the field of aPDT has been on porphyrins, as these are the major class of clinically approved photosensitizers. Recent developments in metalloporphyrins are included herein, however for organic and metalloid porphyrin derivatives see this recent review.^[49]^ Furthermore, the field of nanomaterials for aPDT is flourishing and this area is beyond the scope of this paper, a recent review of this field can be found here.^[50]^ A few papers were not included here as from the data they reported it is unclear if the discussed complexes act through PDT.[^51^, ^52^, ^53^]

### 1.4 Opportunities and challenges in the field

PDT has several key advantages making it particularly suited for the treatment of bacterial infection. This is known as antimicrobial photodynamic therapy (aPDT) or antimicrobial photodynamic inactivation (aPDI). As PDT agents are based on novel and unique chemical scaffolds, preexisting resistance mechanisms based on drug degradation are ineffective. aPDT can also cause bacterial inhibition from outside the bacteria. This can cause damage to key external structures such as membrane bound proteins and cell walls while avoiding resistance through efflux pump overexpression. Furthermore, ROS have no specific cellular target making resistance through changes to target site difficult. ROS have also been shown to damage biofilm biopolymers which are another key resistance mechanism.[^54^, ^55^, ^56^, ^57^] Whether bacteria can develop resistance to ROS through other means remains a matter of debate. Although recent studies by Rapacka‐Zdonczyk *et al*., and Snell *et al*. demonstrated that *S. aureus* could become tolerant to aPDT by a specific PS. This tolerance was not sustained against other PSs indicating that the bacteria did not gain tolerance to the ROS itself.[^58^, ^59^] As the PS must be activated by light, the treatment is limited to external infections or those readily reached with fiber optic equipment. These include dental infections, surface wounds, as well as bladder or lung infections.[^56^, ^60^, ^61^, ^62^] aPDT also has the potential for disinfecting surfaces and destroying biofilms, particularly on biological implants such as catheters where biofilm formation is a serious clinical issue.^[63]^ This represents a large portion of infections including those which might typically be treated with broad‐spectrum antibiotics killing harmful and beneficial bacteria alike. In aPDT, treatment is precisely spatiotemporally controlled preventing such off‐target toxicity.

aPDT does however have some limitations. A drawback of light activation is its ability to penetrate tissue. At shorter higher energy wavelengths biomolecules and water absorb most light meaning the effective depth of treatment is low. The so called “phototherapeutic window” where water and biological molecules are poorly absorbing, is in the red and near infrared (NIR), between approximately 650 and 900 nm. At longer wavelengths light is lower in energy making it harder to excite PSs effectively. Even in the phototherapeutic window, tissue penetration is not more than ∼5 mm.[^64^, ^65^] Due to these intrinsic limitations, aPDT is certainly not a panacea to combat AMR, but it does represent a highly potent tool in the fight against it.

Aside from the limitations of light activation, the field has further challenges to overcome. Ideally, a PS will display selective toxicity towards bacteria over human cells. Preferably this will be due to lack of interaction with, or uptake by, human cells. As the intrinsic characteristics which promote cellular uptake are similar in both eukaryotes and prokaryotes (lipophilicity, positive charge, etc.) creative approaches must be used to promote bacteria specific uptake and prevent off target toxicity. In addition, type II PDT is the most common mode of action for aPDT, however this relies on a supply of O_2_ to generate ROS. Some infections and biofilms are typically hypoxic reducing the application of these PSs. Complexes which can produce type I, or both types of PDT, or combine PDT with other modes of action are therefore highly desirable.

In recent years research into metal complex PSs for aPDT has focused on three main areas: investigation of the effect of the ligands, investigation of the effect of the metal center, and bacteria‐targeted aPDT. In this review, we cover the last five years of progress separated into these three main areas.

## 2 Effect of the Ligands on aPDT

**Figure 2:**
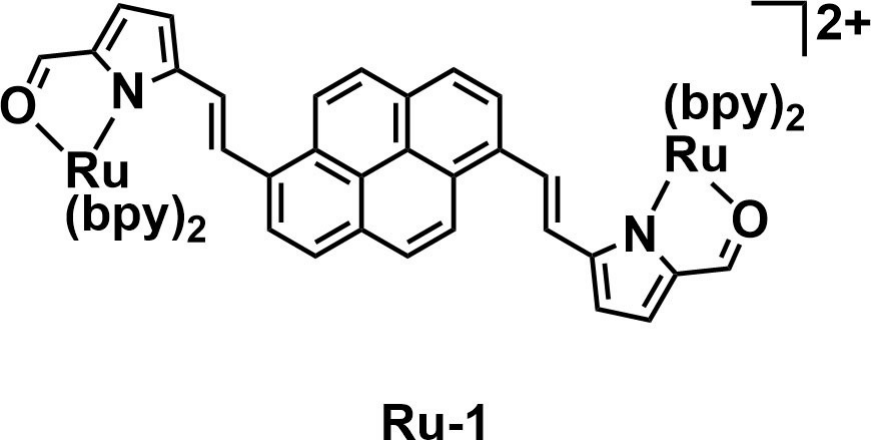
Example from a series of Ru complex trimers by Smithen *et al*. Complex **Ru‐1** contains a perylene core and two ruthenium pyrrole complexes. **Ru‐1** was found to be a potent PS under either white or red (625 nm) light irradiation.

To further their understanding of what makes an effective PS for aPDT, several groups have synthesized series of compounds in which the ligands vary. From such an approach, we can gain insight into how best to tune the photophysical and biochemical properties of a PS. The McFarland group have previously demonstrated that the addition of large aromatic chromophores can greatly increase the potency of PSs.[^66^, ^67^] Building on this, Smithen *et al*. produced a series of ten dinuclear ruthenium complexes capable of acting as PSs for aPDT.^[68]^ These complexes consist of bis[pyrrolyl Ru^II^] triads with organic chromophore cores appended either side with ruthenium pyrrolyl complexes. The series all absorb in the visible region with some absorbing strongly up to 700 nm. The complex with the highest PDT activity (**Ru‐1**, Figure 2) contains a pyrene core and displayed activity under both white and red (625 nm) light irradiation. The phototherapeutic index (PI), sometimes also called the phototoxicity index, is the ratio of light to dark toxicity of a given PS and is often used as a measure of efficacy which can be compared between PSs. Complex **Ru‐1** achieved an extremely high PI>27000 with white light irradiation (100 J cm^−2^) against human promyelocytic leukemia cells (HL‐60). **Ru‐1** was also tested against *Streptococcus mutans* (ATCC 25175) and *S. aureus* (ATCC 25923) in a cell viability assay by serial dilution in a 96‐well plate. 50 % effective concentration (EC_50_) values in the range of 130–160 nM were observed on exposure to white light (100 J cm^−2^), with no toxicity observed in the dark giving PIs >300. With red light (625 nm, 100 J cm^−2^) potency was reduced with PIs >40–50. This work demonstrates a highly effective strategy to achieve red light activated PSs with good activity against *S. mutans* and *S. aureus*.

**Figure 3:**
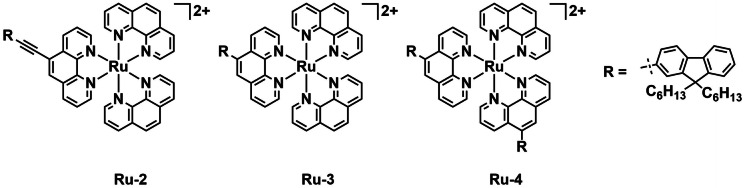
Key examples (**Ru‐2**–**Ru‐4**) from a series of 18 complexes studied as aPDT agents by Le Gall *et al*.

Inspired by the growing global AMR crisis Le Gall *et al*. assembled a series of 18 ruthenium(II) complexes including seven novel examples. The series includes 15 tris‐phenanthroline complexes as well as two bis‐phenanthroline dichloro‐complexes of ruthenium and a bis‐phenylpyridine phenanthroline iridium complex.^[69]^ The complexes were derivatized at the phenanthroline 5‐position with fluorenyl groups, amines, cyano groups as well as one 5‐bromide and one 5,6‐epoxide. The complexes were all assessed for their aPDT efficacy against Gram‐positive *S. aureus* RN4220 and methicillin‐resistant *Staphylococcus aureus* (MRSA) N315 as well as Gram‐negative *Escherichia coli* MG1655, and *Pseudomonas aeruginosa* (PAH). A quick assay was used to give an indication of growth inhibition for all the compounds at 50 μM, both with and without white light irradiation (32 W, 5 min). The complexes with fluorenyl substituents **Ru‐2**, **Ru‐3**, and **Ru‐4** proved to be the most potent PSs (Figure 3) with **Ru‐2** and **Ru‐4** displaying activity against all four strains. To rationalize the complexes’ aPDT activity, their photophysical and chemical properties were investigated. The lipophilicity of the complexes was assessed by determining the butan‐1‐ol/water partition coefficient (log *P*_b/w_).

The complexes containing lipophilic fluorenyl groups had the most positive log *P*_b/w_ values of the series as expected. The ^1^O_2_ generation properties of the complexes were inferred by the degree to which their emission was quenched by O_2_ (using a Stern‐Volmer analysis) the authors theorize that more luminescence quenching would indicate higher ROS generation. Counter to what might be expected, the complexes with the best activity against bacteria displayed less luminescence quenching by O_2_. Uptake of the most potent complex **Ru‐2** by bacterial strains was determined spectroscopically. Bacteria were incubated with the complex and the luminescence intensity was assessed. The bacteria were then separated from the media by centrifugation, and the luminescence of the pellet and supernatant measured to indicate the degree of uptake. The experiments indicate uptake of **Ru‐2** by all strains. Analysis of bacteria treated with **Ru‐2** by cytometry also showed changes in morphology consistent with uptake. The complexes were also investigated for their toxicity against four eukaryotic cell lines (human lung carcinoma (A549), human bronchial epithelial cells (16HBE), human malignant melanoma (SKMEL28), and mouse myoblast cells (C2 C12)), under both white light (32 W, 5 min) and dark conditions. Similar trends observed towards bacteria were reflected in the toxicities towards all the eukaryotic cell lines.

In a separate study by Youf *et al*., complex **Ru‐2** was investigated for its efficacy in an *in vitro* cystic fibrosis model with ruthenium tris‐phenanthroline as a control.^[70]^ **Ru‐2** was further characterized and its ability to produce intracellular ROS was demonstrated by confocal microscopy in both *S. aureus* and *P. aeruginosa* using the standard emissive probe dichloro‐dihydro‐fluorescein diacetate (DCFH‐DA). To investigate how the specific environment of infection in cystic fibrosis can affect aPDT, **Ru‐2** was tested under varied conditions. A reduction in efficacy against bacteria was observed for the complex in a hypoxic environment (low O_2_ levels), as might be expected for a PS which relies on O_2_ to generate ROS, although the effect was modest. Efficacy was also found to be reduced by increased salinity, and decreased pH. The effects of **Ru‐2** on biofilm formation were assessed but found to be modest relative to the control. Finally, genetically modified human bronchial epithelial cells (CFBE‐Luc) were used in conjunction with *P. aeruginosa* to create an *in vitro* infection model. The ability of **Ru‐2** to inhibit bacterial growth, while leaving human cells unharmed was investigated. The viability of the CFBE‐Luc cells was determined from their luminescence. The culture medium was collected and plated to assess bacterial viability by counting colony forming units (CFUs). **Ru‐2** was found to have only a small effect on CFBE‐Luc viability, while under light conditions no bacterial colonies were observed. As in the case of the McFarland groups research, addition of a chromophore gave improved PS activity. Between these two papers a thorough investigation of a series of complexes gave a lead compound with good aPDT efficacy against several clinical strains, and selective activity against *P. aeruginosa* over modified human cells in an *in vitro* model of cystic fibrosis.

**Figure 4:**
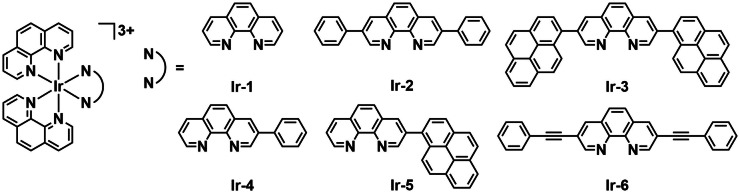
A series of tri‐cationic complexes of iridium developed by Wang *et al*. with ligands containing extended aromatic systems **Ir‐1**–**Ir‐6**.

Wang *et al*. investigated the effect of increased aromatic ligand size on iridium complex aPDT activity. They reported the synthesis and detailed photophysical characterization of six bis‐heteroleptic polypyridyl Ir^III^ complexes (**Ir‐1** to **Ir‐6**, Figure 4) and their activity against *S. aureus* (ATCC 25923) and *S. mutans* (ATCC 25175).^[71]^ These photoluminescent tri‐cationic complexes all bear two phenanthroline ligands. The third ligand is also phenanthroline‐based, but the size of π‐conjugated area varied across the series. All complexes showed long‐lived triplet excited states (>1 μs). **Ir‐3** and **Ir 5** with pyrene substituents displayed ^1^O_2_ quantum yields (*Φ*_Δ_) above 70 % and intense absorption (>450 nm) in the visible light region. Due to these two features, they were both evaluated as PSs for aPDT. The viability was determined using survival assays by measuring the change in bacterial culture turbidity (absorbance at 562 nm). The corresponding dose‐response curves revealed dark toxicities with EC_50_ values of 1–12 μM, while with white light irradiation (35 J cm^−2^) increased toxicities were observed (EC_50_ ∼200 nM) against both *S. aureus* and *S. mutans*. The highest PI of 62 was achieved with complex **Ir‐3** against *S. mutans*. The authors demonstrate further that expansion of the ligands with aromatic moieties achieves enhanced PDT potency.

**Figure 5:**
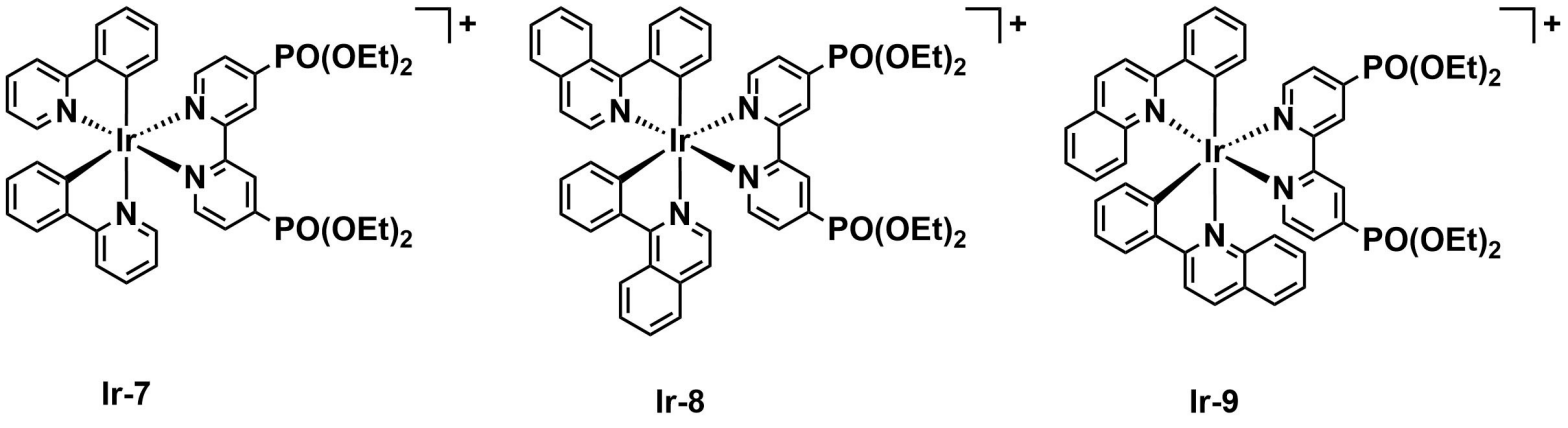
A series of cyclometalated iridium complexes with phosphonate esters **Ir‐7**–**Ir‐9**.

Ho *et al*. reported three cationic cyclometalated Ir^III^ complexes to investigate the effect of the cyclometalating ligand on their aPDT activity.^[72]^ The complexes all feature a bipyridine ligand with two substituted phosphonate esters, while the cyclometalating ligands vary between phenylpyridines and phenylquinolines (**Ir‐7**–**Ir‐9**, Figure 5). These red‐emissive complexes demonstrated enhanced emission intensity in an aggregated state and were found to stain both Gram‐positive and Gram‐negative bacteria by confocal laser scanning microscopy (CLSM). Since the complexes demonstrated decent ^1^O_2_ *Φ*_Δ_ (>30 % in methanol), the authors made use of a CFU‐count method to evaluate their aPDT properties against *Staphylococcus epidermidis* and *E. coli*. (K‐12). Results reveal these complexes are not toxic to bacteria at 5 or 10 μM concentration in dark conditions, except in the case of **Ir‐8** against *E. coli*. With white light irradiation (134 mW cm^−2^, 0.5 h), **Ir‐7** (which has a weak absorption in the mid‐visible light region) was ineffective as a PS. Meanwhile, **Ir‐8** almost entirely eliminates the colony formation of *E. coli* and significantly inhibits the growth of *S. epidermidis* at 10 μM. Although **Ir‐9** had a similar ^1^O_2_ *Φ*_Δ_, it was ineffective as a PS. This study indicates that even relatively small structural changes can greatly affect activity.

**Figure 6:**
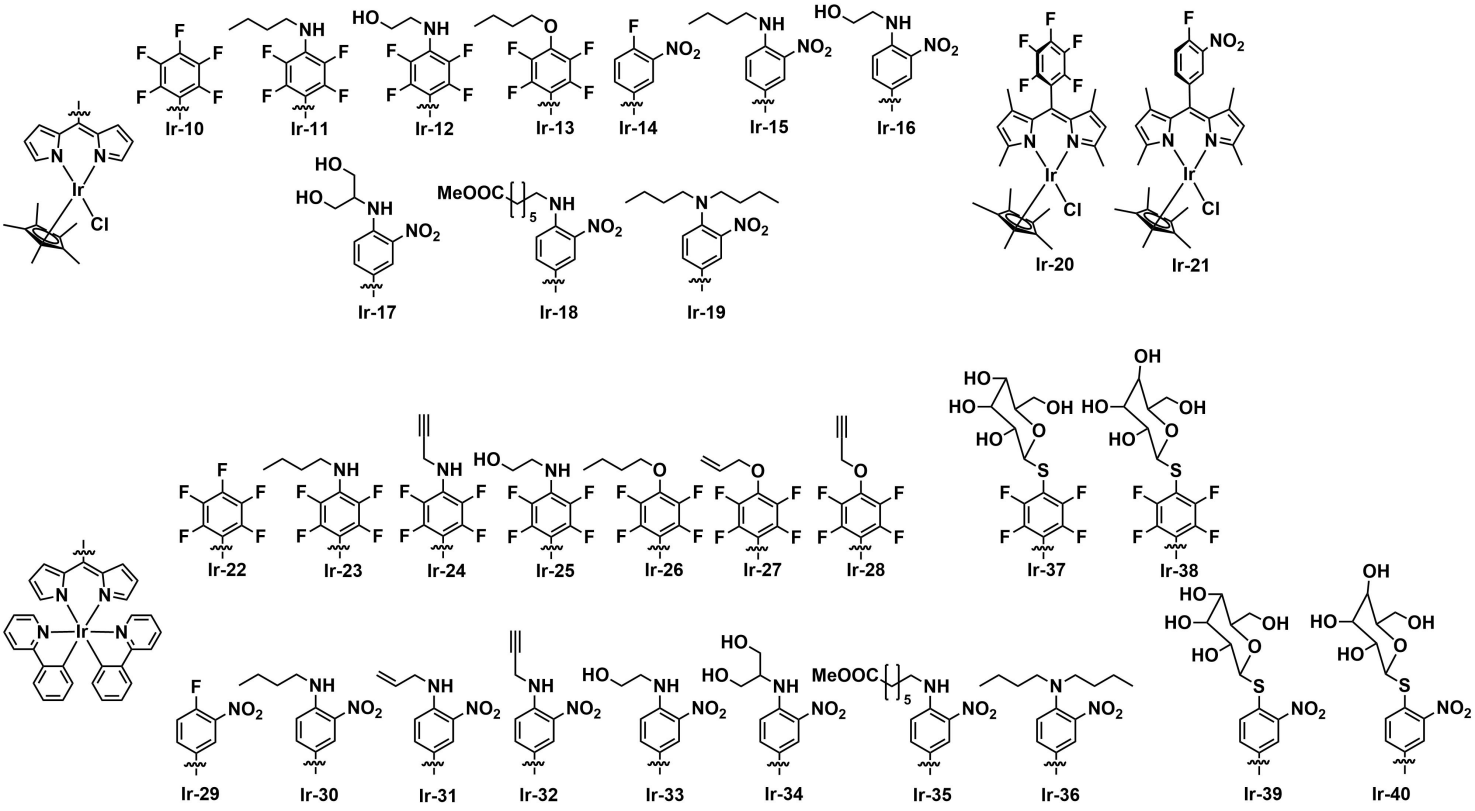
A small library of heteroleptic dipyrrinato‐Ir^III^ complexes (**Ir‐10**–**Ir‐40**) developed by Hohlfeld *et al*.

Hohlfeld *et al*. developed a minilibrary of heteroleptic dipyrrinato‐Ir^III^ complexes for an investigation of structure activity relationship (SAR) on aPDT efficacy.^[73]^ The complexes are divided into chloro(dipyrrinato)(pentamethylcyclopentadienyl) iridium(III) complexes (**Ir‐10**–**Ir‐21**, Figure 6) and bis(2‐phenylpyridyl)(dipyrrinato)iridium(III) complexes (**Ir‐22**–**Ir‐40**, Figure 6). Among these, complexes **Ir‐37**–**Ir‐40** were specifically functionalized with a glucose moiety in the appended phenyl ring using a thioether linkage. In addition to the detailed report of preparation and structural characterization of these derivatives, the authors examined their PDT potencies against human epidermoid cancer (A431), human colorectal adenocarcinoma (HT29). Several complexes were found to be potent cancer PDT agents. aPDT assays were performed on *S. aureus* (ATCC25923) and *P. aeruginosa* (ATCC 27853). The authors compared the bacterial assay results (in CFU measurements) with and without the addition of 10 % serum into the culture media, as the presence of serum rendered a more realistic model for mimicking the *in vivo* environment. For Gram‐positive *S. aureus*, **Ir‐10**–**Ir‐19** (without methyl substitutions on the dipyrrinato ligand) completely eradicate the growth of bacteria at the concentration of 100 μM under dark conditions (without 10 % serum). For Gram‐negative *P. aeruginosa*, complete inhibition did not occur under dark conditions for **Ir‐10**–**Ir‐21** while with light irradiation (white light, 50 J cm^−2^) some examples from the chloro(dipyrrinato)(pentamethylcyclopentadienyl)iridium(III) complexes can entirely inactivate the species at either 10 or 100 μM concentration. Interestingly, the addition of serum reduced the antibacterial potencies of the complexes against both strains. The 2‐phenylpyridyl derivatives (**Ir‐22**–**Ir‐40**) were also investigated against as PSs for aPDT. *S. aureus* was significantly inhibited while the *P. aeruginosa* was not under the same conditions. Moreover, it was found that the glycosylated conjugates **Ir‐37**–**Ir‐40** demonstrated a significant increase in dark toxicity as well as aPDT activity towards *S. aureus*. Furthermore, **Ir‐38** was found to completely inhibit of *S. aureus* at 10 μM under light irradiation in the presence of 10 % serum. This work provides some insight into the effect of structure on aPDT activity. Further studies to determine the reason behind the trends observed would be valuable.

**Figure 7:**
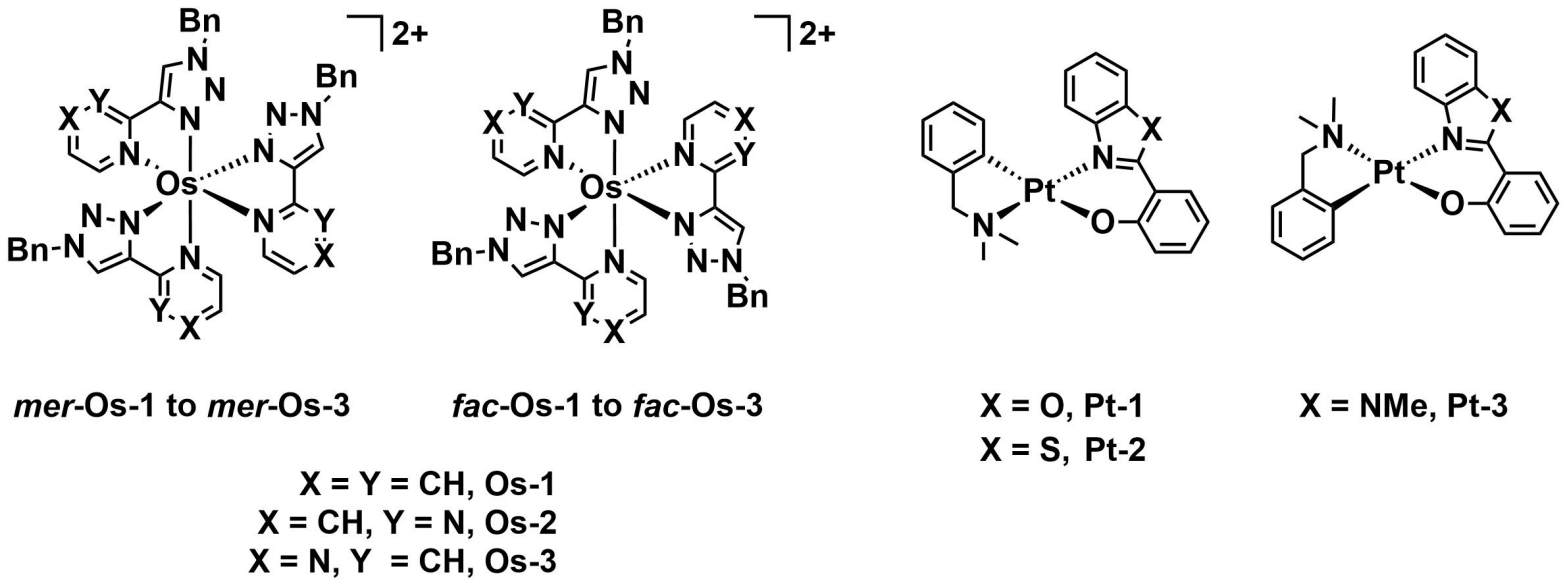
Chemical structures of ***mer***‐ and ***fac*** **‐ Os‐1**–**Os‐3** developed by Ortega *et al*. and 2‐benzoazole‐phenolato platinum complexes **Pt‐1**–**Pt‐3**.

In 2020, Smitten *et al*. reported the synthesis of a series of di‐cationic tris‐homoleptic Os^II^ complexes consisting of triazole‐pyridine, pyrazine and pyrimidine ligands (*mer*‐ and *fac*‐**Os‐1**–**Os‐3**, Figure 7).^[74]^ The complexes displayed absorption maxima from 480 to 530 nm and emission bands lie in the deep red/near‐infrared region. Super‐resolution imaging studies demonstrated the complexes were taken up by MRSA and primarily located in the DNA of the bacteria. Uptake was further investigated in MRSA and *E. coli* (EC598) by inductively coupled plasma atomic emission spectrometry (ICP‐AES). The results indicate that ***mer*** **‐Os‐1** was taken up more than ***fac*** **‐Os‐1** in both strains, while both isomers were more strongly taken up by MRSA than *E. coli* minimum inhibitory concentration (MIC) assays were employed to investigate whether the complexes inhibited the growth of *E. coli*, MRSA, *Acinetobacter baumannii* (AB184), and *P. aeruginosa* (PA2017). ***mer*** **‐Os‐1** and ***mer*** **‐Os‐2** demonstrated the lowest MIC values against MRSA, of 32 and 64 μg/mL respectively. However, the growth of the other bacterial species was not inhibited by any of the complexes. The *mer*‐congeners were further studied to determine their phototherapeutic effects towards MRSA with exposure to broadband light (48 J cm^−2^), but this only rendered a slightly enhanced inhibition activity with a phototherapeutic index of ∼2. In addition, the *mer* isomers of these metal complexes showed a more potent antibacterial efficacy over the corresponding *fac* isomers likely due to their difference in uptake. In this study the importance of uptake on activity is demonstrated, as well as the difference in activity of stereoisomers, although the PDT effects are limited.

Three square‐planar cyclometalated Pt^II^ complexes (**Pt‐1**–**Pt‐3**, Figure 7) were recently reported by Ortega *et al*., and these neutral luminescent complexes were investigated as antibacterial and anticancer agents.^[75]^ These complexes all contain a cyclometalated *N*,*N*‐dimethylbenzylamine ligand and vary with regard to their 2‐benzoazole‐phenolato N−O ligands. Two Gram‐positive and two Gram‐negative bacteria from the notorious ESKAPE group (vancomycin‐resistant *Enterococcus faecium*, MRSA, *A. baumannii*, and *P. aeruginosa* were chosen in order to examine the antibacterial properties of these complexes with and without light treatment. **Pt‐1** and **Pt‐3** did not exhibit strong antibacterial activity against Gram‐negative species, whilst **Pt‐2** exhibited a significant inhibition of *E. faecium* and *MRSA* under dark conditions with MIC values of 5 and 30 μM, respectively. Upon blue light irradiation (420 nm, 40 min, 6 J cm^−2^), the MIC values further decreased for both Gram‐positive strains. **Pt‐2** achieved a PI of 15 against MRSA. Here, a minor change in heteroatom in the N−O ligand led to a significant change in aPDT effect.

**Figure 8:**
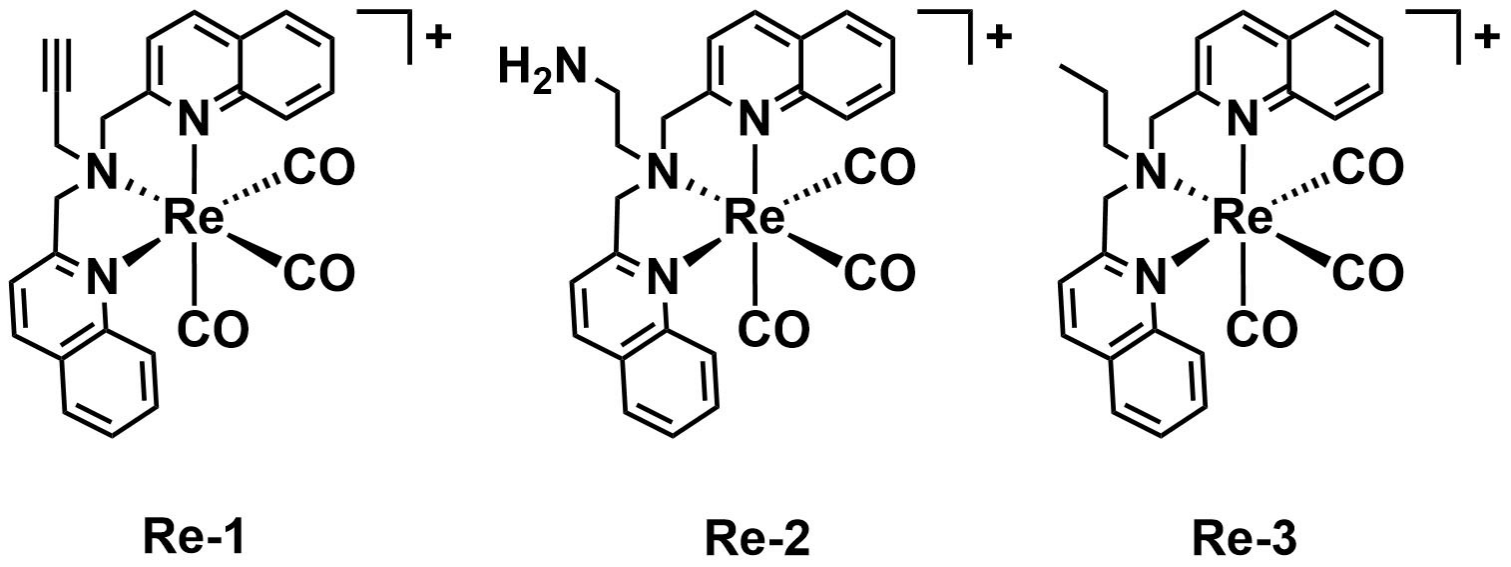
The Re‐based PSs for aPDT **Re‐1**–**Re‐3** reported by Frei *et al*.

Inspired by previous reports of rhenium antimicrobials, Frei *et al*. prepared rhenium‐based complexes for aPDT. They found that the complexes inhibit the growth of Gram‐positive and Gram‐negative bacteria.^[76]^ The bisquinoline scaffold with a tertiary amine was chosen as the tridentate ligand with either alkyne, amine, or alkyl groups (**Re‐1**–**Re‐3**, Figure 8). Their inhibition potencies against a variety of Gram‐positive and Gram‐negative bacterial strains were examined using MIC assays performed in the dark and under UV‐light (365 nm, 3 J cm^−2^). The complexes obtained nano‐ to micromolar MIC values revealing that they can inhibit the growth of *S. aureus* both with and without light irradiation. For the case of *E. coli*, no antibacterial activity was observed in the dark while micromolar MIC values were achieved under light irradiation. Furthermore, the most active complex, **Re‐1** also showed aPDT activity against drug resistant bacteria (MRSA and mobilized colistin resistant *E. coli*). Using ICP‐MS the authors showed that activity of **Re‐1** against different strains correlates with its uptake.

**Figure 9:**
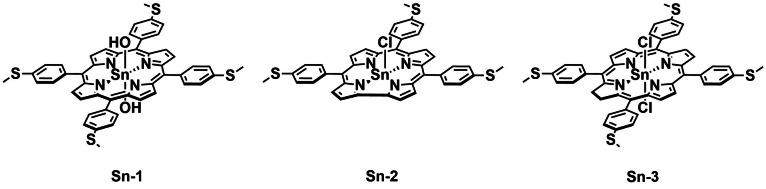
Red light‐activated macrocyclic tin‐based PSs **Sn‐1**–**Sn‐3**.

Dingiswayo *et al*. reported the preparation of Sn^IV^‐based porphyrin, corrole, and chlorin complexes with 4‐methylthiolphenyl moieties linked to the macrocycle rings to investigate which macrocycle yields the best PS (**Sn‐1**–**Sn‐3**, Figure 9).^[77]^ The complexes were assessed for their PDT activities against bacteria as well as cancer. To determine the effect of Sn‐coordination, the free‐base precursors were compared to the Sn‐complexes throughout. In addition, the metal complexes displayed enhanced ^1^O_2_ *Φ*_Δ_ (*>*0.48) compared to the free ligands. As **Sn‐3** and its free‐base precursor exhibited the best PDT activity in cancer cells, they were also investigated for their aPDT efficacy. **Sn‐3** and its precursor displayed insignificant dark toxicity towards both *S. aureus* (ATCC 25923) and *E. coli* (ATCC 25922). After 30 min red light irradiation (660 nm, 280 mW cm^−2^), **Sn‐3** achieved complete inhibition of both *S. aureus* and *E. coli*. The free ligand was, however, found to be ineffective as a PS against *E. coli*, even with 60 minutes’ irradiation. This demonstrates that combining a chlorin macrocycle with the heavy metal Sn can achieve efficient red light‐activated ROS generation and aPDT activity in Gram‐negative and Gram‐positive bacteria.

**Figure 10:**
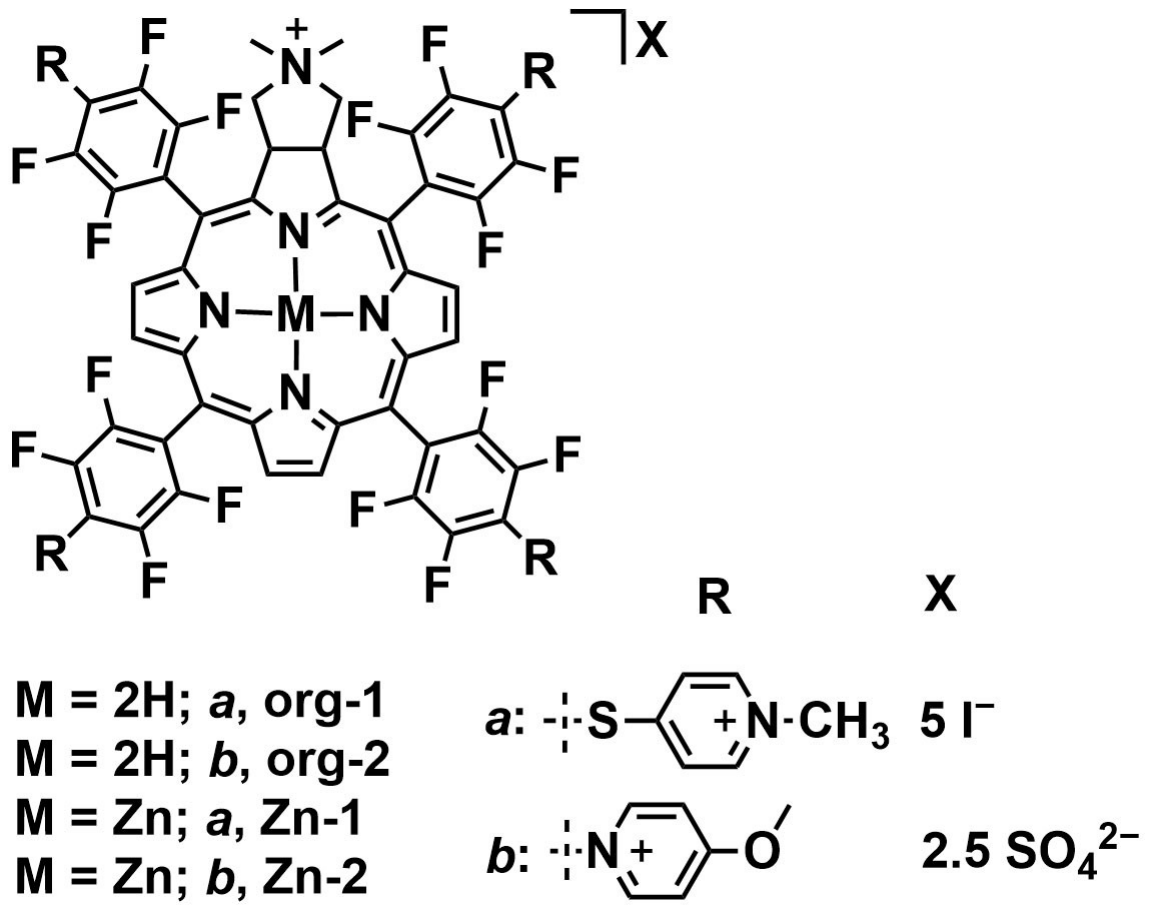
Chemical structures of **Zn‐1**, **Zn‐2** and their chlorin precursors **org‐1** and **org‐2**.

Calmeiro *et al*. were also interested in metallochlorins as PSs and developed zinc complexes with *N*‐methylpyridinium or methoxypyridinium substituents (**Zn‐1** and **Zn‐2**, Figure 10).^[78]^ The group investigated the effect of the pyridinium charge location on the PS efficacy. The metal complexes displayed improved ^1^O_2_ *Φ*_Δ_ compared to their chlorin counterparts (**org‐1** and **org‐2**, Figure 10) and the highest value of 0.63 was reached by **Zn‐2**. The complexes and ligands were assessed as PSs for aPDT against bioluminescent *E. coli*. Viability was determined by change in bioluminescence. Under dark conditions negligible toxicity was observed for all four compounds. With white light irradiation (25 mW cm^−2^, 0–120 min), **Zn‐1** was found to be the best PS among the four compounds and achieved 5.2 log reduction of bioluminescence after 45 min of light irradiation. In this instance, we see that although **Zn‐2** was the most efficient PS in terms of ^1^O_2_ *Φ*_Δ_ this did not translate to better efficacy against *E. coli*. The type of pyridinium substituent is clearly important as ROS generation is not a factor. It may therefore be inferred that uptake plays a role in this case although the authors do not investigate this further.

**Figure 11:**
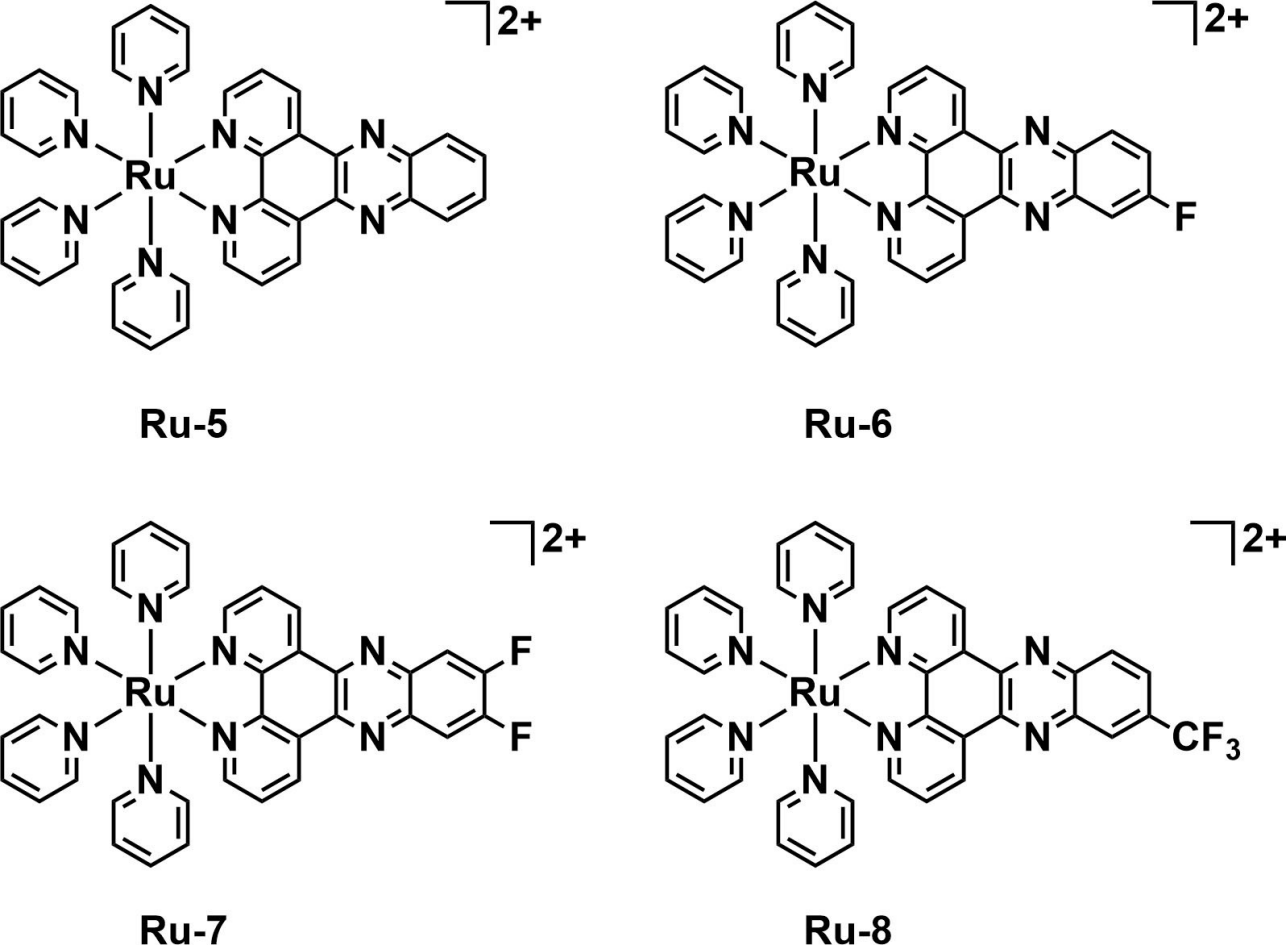
A series of ruthenium complexes with fluorinated dppz bidentate ligands and pyridine monodentate ligands as DNA intercalating aPDT agents by Sun *et al*.

Sun *et al*. synthesized a series of dipyrido[3,2‐*a*:2’,3’‐*c*]phenazine (dppz) complexes of ruthenium(II) in order to take advantage of the ligand's DNA intercalating properties (**Ru‐5**–**Ru‐8**, Figure 11).^[79]^ All the complexes are increasingly fluorinated across the series at the 11‐ and 12‐positions of the dppz ligand. In addition, the complexes all contain four pyridines as photolabile monodentate ligands. The complexes were tested for their activity against *S. aureus*, with and without blue light irradiation (470 nm) to determine both MIC and minimum bactericidal concentration (MBC) values utilizing methicillin and vancomycin as controls. The complexes all displayed no inhibition of bacterial growth in the dark and significant bacteriostatic and bactericidal properties under irradiation with blue light against all four strains. **Ru‐8** was the most potent with MBCs of 8, 1, 2 and 4 μM against *E. coli*, *S. aureus*, MRSA, and vancomycin‐resistant Enterococcus (VRE) respectively. The experiments were repeated in hypoxic conditions and found to yield the same results indicating an O_2_‐independent mode of action. The ^1^O_2_ generation properties of the complexes were investigated spectroscopically with 9,10‐ADPA as a ^1^O_2_ trap and [Ru(bpy)_3_]^2+^ as a known standard. All the complexes displayed only modest to poor ^1^O_2_ generation properties. The toxicity of pyridine and the photodissociation product of **Ru‐8** were tested in the dark and while pyridine was shown to have very little inhibitory effect, the photodissociation product retained the same efficacy as the parent complex under light conditions. As dppz complexes of Ru^II^ are known to intercalate with DNA, the DNA interaction of the complexes was studied along with the uptake by *S. aureus* cells using ICP‐MS. The complexes showed increasing levels of uptake and DNA binding with fluorination matching the trend in toxicity. This may not wholly be explained by lipophilicity as the octanol/water partition coefficients (log *P*_o/w_) are similar across the series (−1.09 to −1.15). The authors postulate that fluorine plays another role in uptake such as facilitating hydrogen bonding interactions. The complexes were also tested against rabbit blood cells and L‐02 (human hepatocytes) to assess their toxicity to mammalian cells. Low levels of hemolysis were observed in rabbit blood cells and the half maximal inhibitory concentration (IC_50_) values of the complexes **Ru‐7** and **Ru‐8** against L‐02 were all in excess of 200 μM in the dark. With light irradiation (470 nm) at 5 μM concentration of **Ru‐7** and **Ru‐8**, 70 % and 90 % of L‐02 cells survived after 24 h, respectively. The selectivity of **Ru‐8** was further investigated by coincubation of L‐02 cells with *S. aureus* in 5 μM **Ru‐8**. After 4 h the cells were treated with propidium iodide and imaged by CLSM. Only the bacterial cells showed the red emission indicative of propidium iodide uptake and cell death. The complexes were shown to be effective PSs with the dual functions of ROS and photorelease induced‐DNA binding as well as selectivity towards *S. aureus* over L‐02 cells. The authors demonstrate that tuning of the physiochemical properties of a PS by fluorination can be an effective strategy.

**Figure 12:**
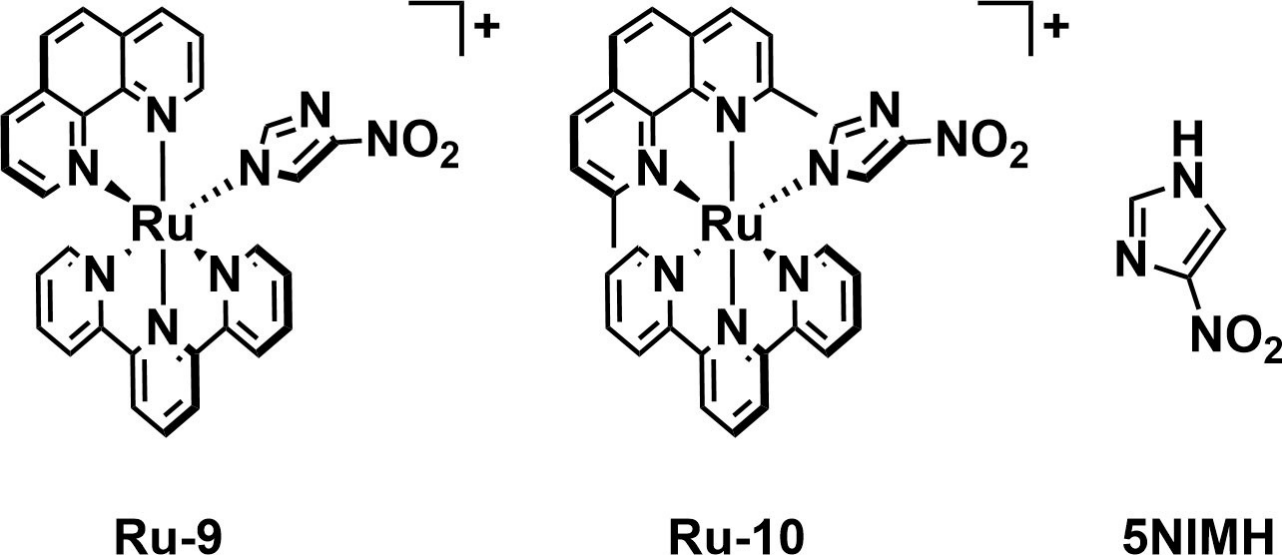
Nitroimidazole‐based ruthenium complex **Ru‐10** which can undergo photosubstitution with blue light (434 nm) irradiation as well as controls **Ru‐9** and **5NIMH**.

In order to combine the strategies of photorelease and PDT, Giacomazzo *et al*. synthesized a pair of tridentate, tris‐heteroleptic ruthenium complexes (**Ru‐9** and **Ru‐10**, Figure 12).^[80]^ Both complexes contain phenanthroline, terpyridine, and nitroimidazole ligands. Complex **Ru‐9** differs from **Ru‐10** with methyl groups at the phenanthroline 2‐ and 9‐positions. The addition of these methyl groups give rise to distortion of the octahedral structure of **Ru‐10**. This distortion causes the ejection of nitroimidazole from **Ru‐10** on irradiation with blue light (434 nm), while **Ru‐9** without methyl groups is photostable. Differential pulse voltammetry (DPV), ultraviolet‐visible spectroscopy, liquid chromatography mass spectrometry (LC–MS), and high‐pressure liquid chromatography (HPLC) were utilized to verify this behavior. The complexes along with 5‐nitroimidazole (**5NIMH**, Figure 12) were tested against *Bacillus subtilis* 168 to determine their effects on bacterial cell viability in both dark and light (434 nm, 160 mW) conditions. No dark toxicity was observed for **5NIMH** or **Ru‐9** except at 500 μM, while **Ru‐10** displayed a dose‐dependent toxicity in the dark. Under irradiation, no appreciable change was observed for **5NIMH** or **Ru‐9**, while **Ru‐10** showed a clear enhancement of activity. The photosubstituted product of **Ru‐10** in water, ([Ru(tpy)(dmp)(H_2_O)]^2+^), was also tested for its PDT efficacy and this showed lower activity than **Ru‐10**. The authors suggest that the enhanced toxicity of **Ru‐10** is due to the ROS generation properties of **5NIMH**. **5NIMH** itself is not efficacious as a PS in this instance making **Ru‐10** greater than the sum of its parts.

## 3 Effect of the Metal Center on aPDT

**Figure 13:**
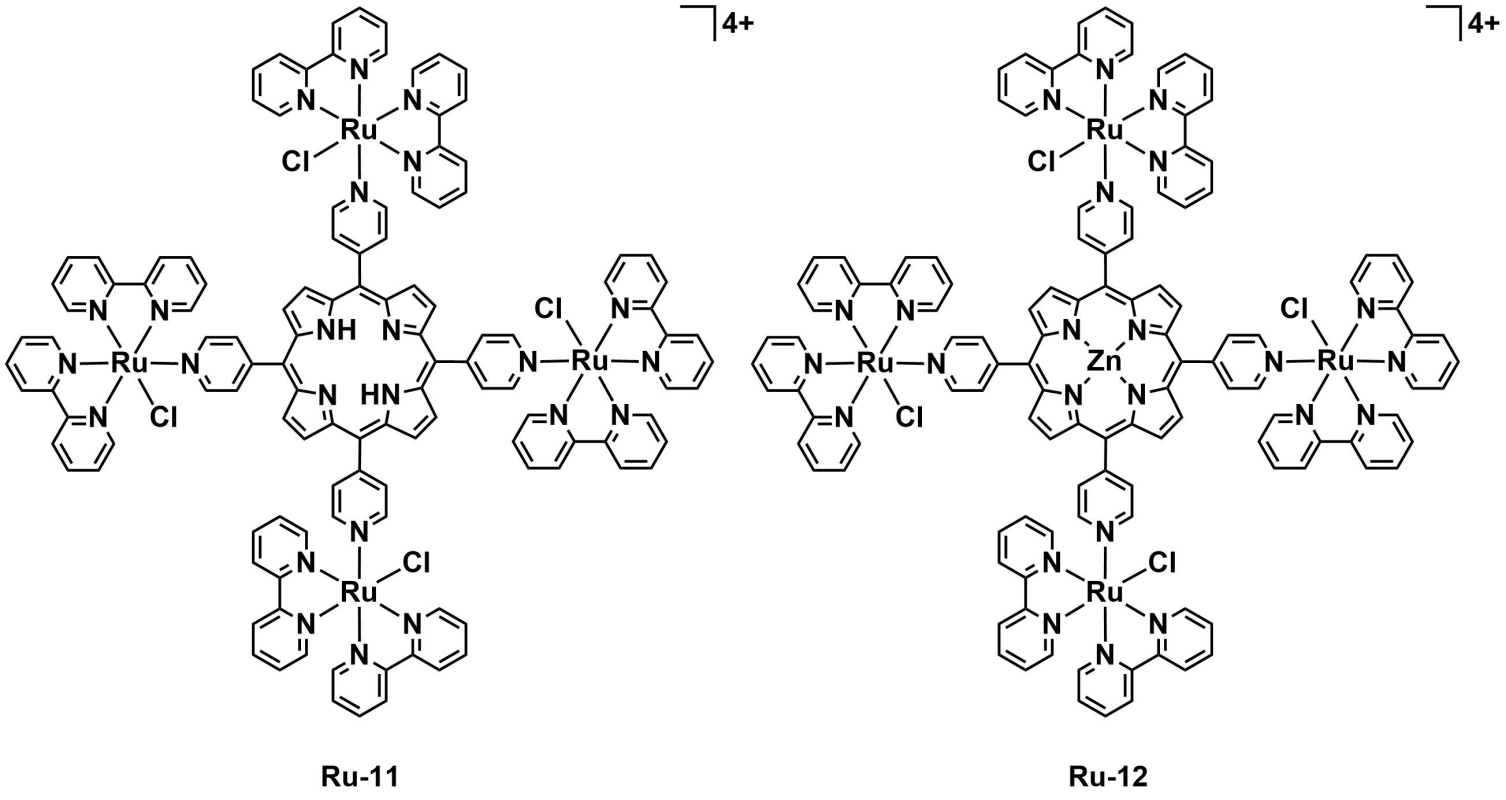
A pair of polynuclear complexes of ruthenium by Gonçalves *et al*. **Ru‐11** and **Ru‐12** which can generate ^1^O_2_, O_2_^⋅−^, and HO^⋅^ to kill bacteria.

**Figure 14:**
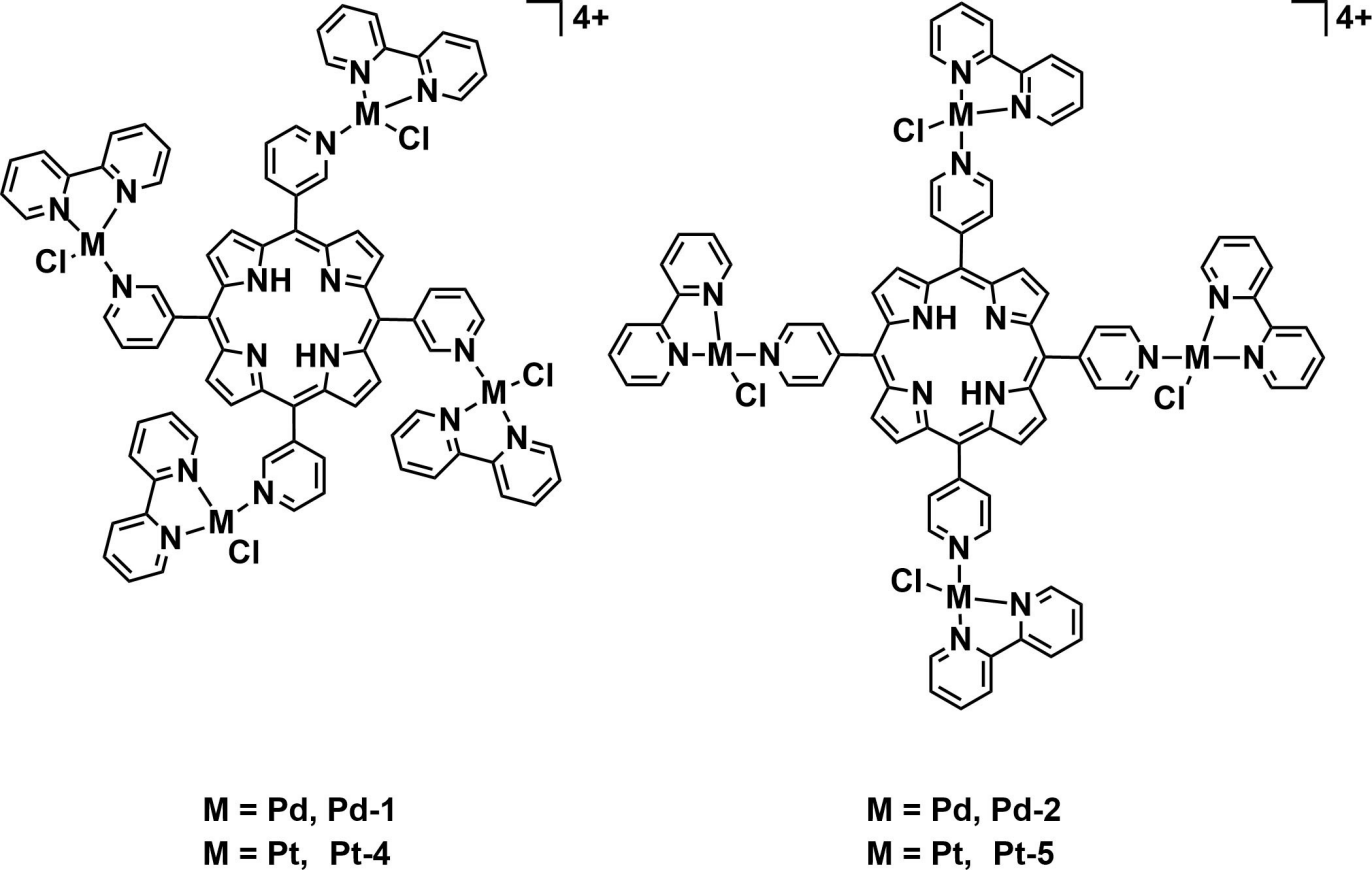
Palladium‐ and platinum‐complex‐derivatized porphyrins **Pd‐1**, **Pd‐2**, **Pt‐3**, and **Pt‐4**.

Another common subject of investigation in recent work on aPDT is the variation of the metal center. By judicious selection of the metal center, significant changes in absorption, emission, charge, and ROS generation can be achieved. The Iglesias group was interested in combining two potent types of PS (metal complexes and porphyrins) to develop a new class of complex for aPDT. The group published a series of papers in which the metals are varied to study the effect of this on aPDT efficacy. Gonçalves *et al*. developed a ruthenium and zinc bimetallic system, which upon activation by white light generates ^1^O_2_ as well as O_2_^⋅−^ and HO^⋅^ to kill *Salmonella enterica* serotype *typhimurium*.^[81]^ The complex consists of a porphyrin core appended with four Ru complexes. Both the uncoordinated porphyrin and zinc porphyrin complexes (**Ru‐11** and **Ru‐12**, Figure 13) were tested against *S. enterica* and while no toxicity was observed in the dark, with 30 min irradiation (360 J cm^−2^) no bacterial growth was observed for both complexes. Detailed study of the ROS species generated by the complexes was performed using electron paramagnetic resonance (EPR) spectrometry. Different radical species generate a unique fingerprint in EPR and can thereby be identified. The authors discovered that their complexes not only generate ^1^O_2_, but also O_2_^⋅−^, and HO^⋅^ making them potent PSs. The author's thorough investigation of the ROS generated demonstrates an important avenue for aPDT, which is often overlooked. An isomeric pair of tetra‐cationic porphyrins with four peripheral [Pd^II^(bpy)Cl]^+^ moieties (**Pd‐1** and **Pd‐2**, Figure 14) were also prepared by the group and investigated for aPDT against four bacterial strains: *S. aureus* (ATCC 6853), *E. coli* (ATCC 25922), *P. aeruginosa* (PAO1), and *Klebsiella pneumoniae* (ATCC 1705).^[82]^ Structurally, the isomers originate from the peripheral pyridine rings’ substitution position on the centered porphyrin ring. With this subtle change, the ^1^O_2_ *Φ*_Δ_ improved from 0.26 to 0.51 from the *para*‐ to *meta*‐pyridine substituted complexes, respectively. In addition, ^1^O_2_ *Φ*_Δ_ values were greatly improved compared to the pure organic precursors. MIC assays against the abovementioned microorganisms were carried out under dark and light irradiation conditions (400–800 nm, 270 J cm^−2^). It was found that the addition of Pd complexes to the porphyrin core ameliorated inhibition in both dark and light conditions for all the tested bacteria. Photoinactivation was likely enhanced due to the greatly increased ^1^O_2_ *Φ*_Δ_. **Pd‐1** was found to be the most active PS against *S. aureus* and *K. pneumoniae* with MICs of 2.00 μg mL^−1^. The same complexes (**Pd‐1** and **Pd‐2**) were employed to inhibit the growth of four mycobacteria strains, (*Mycobacteroides ab*scessus, *Mycolicibacterium fortuitum*, *Mycobacterium smegmatis*, and *Mycobacteroides abscessus* subsp. *Massiliense*).^[83]^ MIC assays were used to investigate the photoinhibition activity of the tetra‐cationic species under dark and white light (270 J cm^−2^) conditions. The most potent photosensitizer **Pd‐1** gave MIC values between 1.21 and 2.43 μM under light irradiation and 39 μM in dark conditions for all the abovementioned strains. Atomic force microscopy (AFM) investigation revealed that the Pd^II^‐based photosensitizer damaged the mycobacterial cell wall upon light exposure.

**Figure 15:**
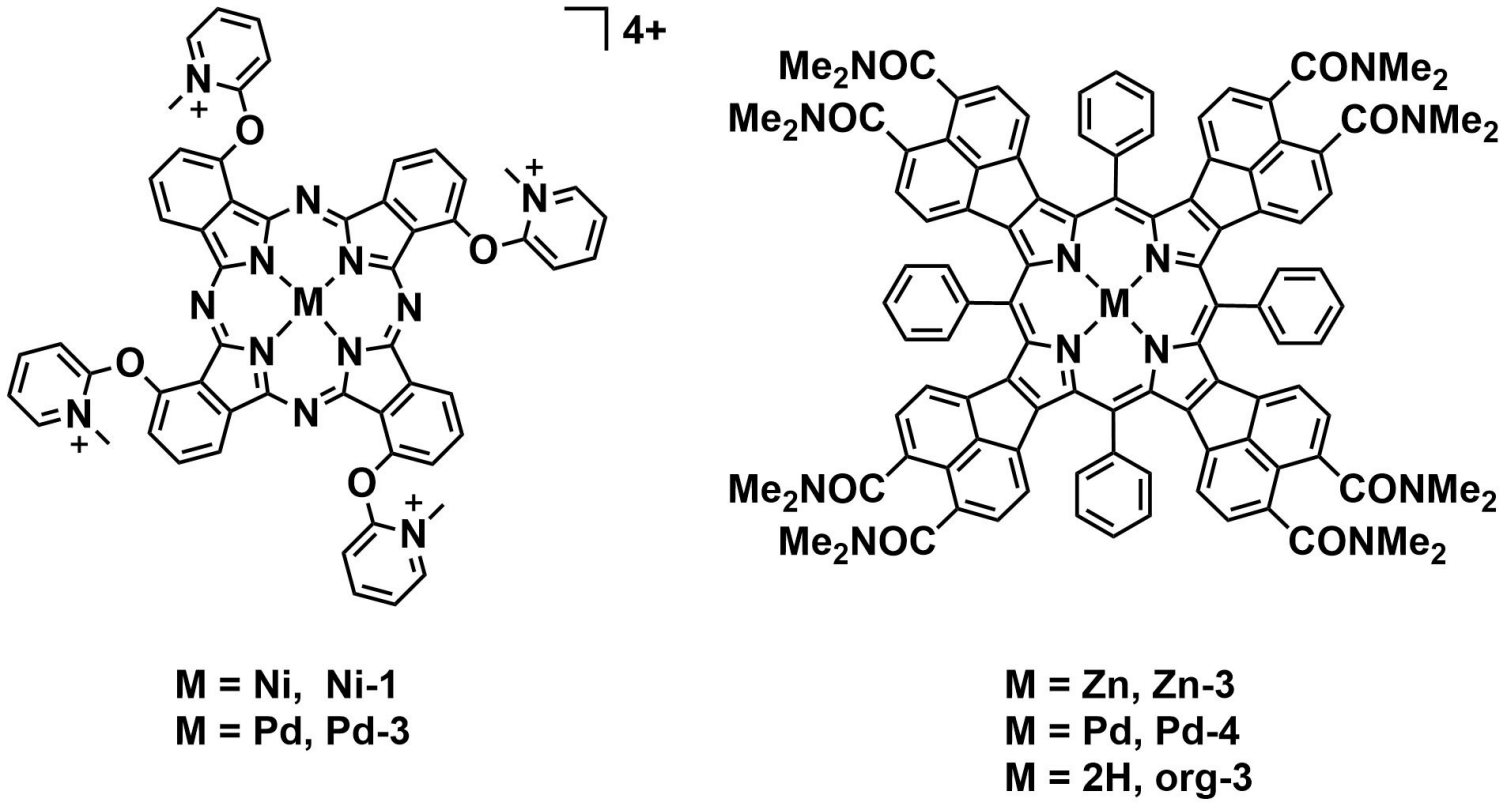
Phthalocyanine‐based PSs **Ni‐1** and **Pd‐3** as well as metalloporphyrins **Zn‐3**, **Pd‐4** and organic precursor **org‐3**.

The Iglesias group went on to investigate **Pt‐4** and **Pt‐5** complexes, the Pt derivatives of **Pd‐1** and **Pd‐2** (Figure 14) and evaluated their photoinactivation performance against the same four mycobacteria strains.^[84]^ In their study, the authors also compared the pure organic precursors with the two isomeric Pt^II^‐containing complexes in terms of their photodynamic inhibition efficacy against the mycobacteria. Under dark conditions, all the chemical species reported the same MIC value of 93.75 μg mL^−1^ against the four microorganisms, whereas the complex with *meta*‐position substituted peripheral moiety **Pt‐4**, possessed the lowest MIC value (0.36 to 1.76 μg mL^−1^) against the four mycobacteria upon light irradiation (400–800 nm, 270 J cm^−2^). The two organic compounds did not exhibit significant variation of MIC values between dark and light conditions. The Iglesias group investigated variation of both metal center and structural isomers on PDT efficacy. Both aspects were found to affect the photophysical properties and thus the potency of photosensitization. Kulu *et al*. synthesized tetra‐cationic Ni^II^‐ and Pd^II^‐coordinated phthalocyanines complexes with four methyl pyridinium substituents (**Ni‐1** and **Pd‐3**, Figure 15) to determine the effect of the metal center on aPDT efficacy.^[85]^ These complexes were investigated for their bacterial uptake capability and photodynamic activity at 5 and 10 μM against two pathogenic bacterial strains: Gram‐positive MRSA (1337 Collection of the Institute of Microbiology, Bulgarian Academy of Sciences, Sofia), and Gram‐negative *Aeromonas hydrophila*. The variation in metal centers did not have a significant effect on the complexes’ UV/Vis absorption spectra but rendered a huge difference in their singlet oxygen quantum yields. **Ni‐1** possesses a ^1^O_2_ *Φ*_Δ_ of 0.01 in aqueous solution while the **Pd‐3** has a ^1^O_2_ *Φ*_Δ_ of 0.26 in the same conditions. These differences were ascribed to increased intersystem crossing (ISC) and spin‐orbit coupling capability due to the heavy atom effect. Uptake of the complexes was determined spectroscopically and it was also found that **Pd‐3**, was more efficiently internalized by the two bacterial species than **Ni‐1** by one order of magnitude. Upon red‐light irradiation (660 nm, 60 J cm^−2^), both **Ni‐1** and **Pd‐3** did not effectively inactivate the growth of *A. hydrophila*. Under the same conditions **Ni‐1** and **Pd‐3** were able to inhibit MRSA, with **Pd‐3** displaying the most aPDT potency. Here, Kulu *et al*. successfully demonstrate the enhancement of photosensitization by choice of the correct metal center. In 2020, Galstyan *et al*. reported the synthesis of π‐extended organic porphyrin (**org‐3**, Figure 15) and metalloporphyrins (**Zn‐3** and **Pd‐4**, Figure 15) as PSs for aPDT.^[86]^ The porphyrin and metalloporphyrins exhibited NIR absorption due to the extended π‐conjugation of the fused naphthalene units. Additionally, dimethyl amide substituents were added to aid their aqueous solubility. Critically, ^1^O_2_ *Φ*_Δ_ values in water were found to be 0.07, 0.39, and 0.50 for **Zn‐3**, **Pd‐4**, and **org‐3**, respectively, using methylene blue (MB) as the reference. Two Gram‐positive bacteria *S. aureus* (3150/12) and *B. subtilis* (DB104) were employed in the PDT assays (515 nm, 5 W cm^−2^, 30/60/90 min). **Pd‐4** was found to be the most efficient photosensitizer, with almost complete inhibition of *S. aureus* and >3 log_10_ reduction of *B. subtilis* at the highest dose of 27 J cm^−2^. Compared to **Pd‐4**, **Zn‐3** and **org‐3** were less effective PSs. The authors suggest that the markedly higher photocytotoxicity of **Pd‐4** implies a higher cellular uptake as supported by the higher log *P*_o/w_. In addition, **Pd‐4** displayed reduced efficacy in the presence of mannitol (a HO^⋅^ scavenger), which implies the complex could act through both type I and type II PDT mechanisms. In addition, we see the same trend as in the previous work where the heavier metal gives higher ^1^O_2_ *Φ*_Δ_.

## 4 Bacterium‐Targeted aPDT

In order to address the issue of selective toxicity towards bacteria over human cells, several groups have investigated strategies for selective uptake and/or interaction with bacteria. The recent examples are all ruthenium complexes but utilize a variety of different approaches to yield excellent selectivity. This demonstrates the potential of aPDT to be a highly targeted modality. Given the negative charge of bacterial cell walls and outer membranes Feng *et al*. developed a series of highly positively charged PSs which are specifically taken up by MRSA over mammalian cells and display aPDT activity on irradiation with blue light (470 nm).^[87]^ The complexes are based on a ruthenium tris‐bipyridine ([Ru(bpy)_3_]^2+^) core with increasing numbers of trimethyl ammonium groups thus leading to increasing overall positive charge of the complex from +2 to +8. The complexes were assessed for their ability to generate ^1^O_2_.

**Figure 16:**
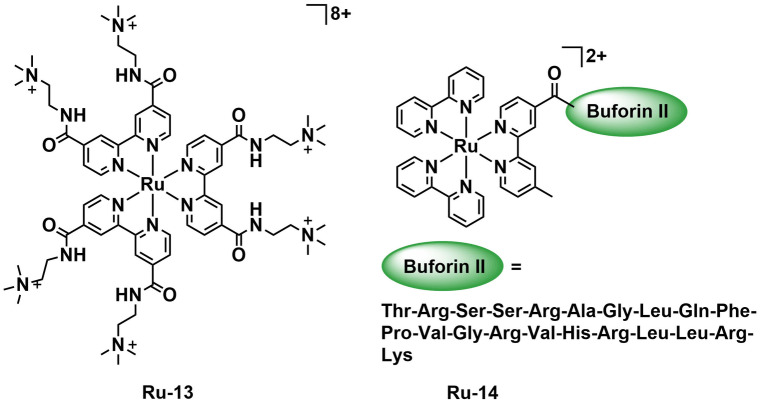
Example of an octacationic ruthenium complex with specific toxicity towards MRSA over mammalian cells by Feng *et al*. (**Ru‐13**) and **Ru‐14** a complex attached to a DNA‐targeting peptide as a bacteria selective PS for aPDT.

Their ^1^O_2_ quantum yields (*Φ*_Δ_) were determined spectroscopically using 3,3’‐(anthracene‐9,10‐diyl)dipropanoic acid (9,10‐ADPA) as a ^1^O_2_ trapping agent and ruthenium tris‐bipyridine ([Ru(bpy)_3_]^2+^) as a known standard. Complex **Ru‐13** (Figure 16) was found to have a ^1^O_2_ *Φ*_Δ_ of 0.38±0.04 similar to that of [Ru(bpy)_3_]^2+^ (0.41). *S. aureus* treated with the complexes in the dark showed no inhibition, but with exposure to 470 nm light, dose‐dependent inhibition was observed. The complexes also displayed activity against MRSA on exposure to light although the inhibitory effect was reduced compared to that against *S. aureus*. Complex **Ru‐13** was the most potent PS in both cases showing a 6.87 and 5.75 log reduction of colony forming units at 15 μM concentration against *S. aureus* and MRSA, respectively. The complexes also displayed modest efficacy against *E. coli*. Uptake of the complexes was measured using inductively coupled plasma mass spectrometry (ICP‐MS) to analyze the Ru content of the cells. Complex **Ru‐13** was shown to have the greatest uptake with 25.40 and 27.55 ng Ru/10^8^ cells in *S. aureus* and MRSA respectively, more than 8‐fold that of the other complexes. The toxicity of the complexes towards mammalian cells was investigated against HeLa (human uterine carcinoma), 293T (human embryonic kidney transformed 293 cells), L‐02, and rabbit red blood cells. In all cases low toxicity was observed with around ∼80 % retention of viability of HeLa cells for all complexes on exposure to light (470 nm). A cocultured sample of *S. aureus* and 293T was treated with light, **Ru‐13**, and propidium iodide. Red emission indicating cell death was observed only in the bacteria and not in the human cells indicating the selectivity of **Ru‐13** as a PS. Overall, Feng *et al*. demonstrate an interesting and effective strategy to achieve selective aPDT towards bacteria over human cells.

Buforin II is an antimicrobial peptide (AMP), which displays broad spectrum antibiotic activity, able to enter bacteria and interact with DNA to cause cell death. Pierce *et al*. synthesized a [Ru(bpy)_3_]^2+^ complex (**Ru‐14**, Figure 16) attached to Buforin II via the N terminus, and were able to enhance the efficacy of the AMP adding light activated ^1^O_2_ generation to its antimicrobial properties.^[88]^ **Ru‐14** was tested against *E. coli* (MG 1655) and *B. subtilis* (1 A1) achieving MIC values of 32 and 16 μM in the dark respectively. On blue light (470 nm, 12 mW cm^−2^, 12 h) irradiation, a 32‐fold increase in toxicity was observed against *E. coli* as well as an 8‐fold increase against *B. subtilis*. It should be noted some increase in toxicity was observed even with controls indicating the irradiation parameters may have been too high. To investigate the type of ROS generation involved, the MIC experiments were repeated with scavengers for O_2_^⋅−^, HO_2_^⋅^, and HO^⋅^ species and no changes were observed, indicating ^1^O_2_ generation may be the cause of the PDT effect. The efficacy of **Ru‐14** was further examined against a range of multidrug resistant clinical isolates: *P. aeruginosa* AR0229, *E. coli* AR0114, *A. baumannii* Naval‐17, and *K. pneumoniae* AR0113. Similar efficacy was observed as for the previous experiment with MICs ranging from 0.5–4 μM with light irradiation, and increases in toxicity from eight‐ to 32‐fold. **Ru‐14** was proven to be toxic against an array of clinical isolates, however, the high dark toxicities reduce the selectivity of the complex and further work on the toxicity towards eukaryotes is needed.

**Figure 17:**
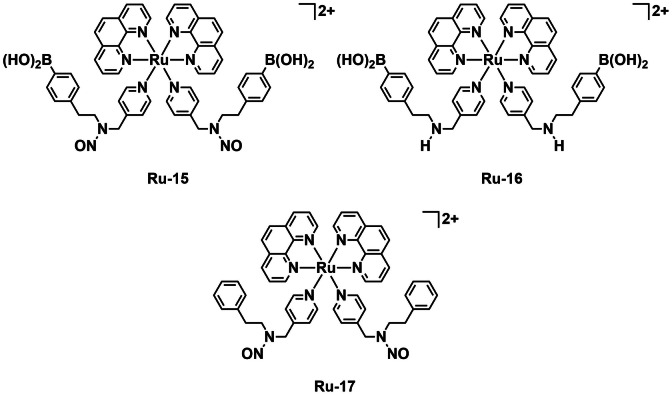
A bimodal ruthenium complex for the treatment of bacterial biofilms (**Ru‐15**) that generates ^1^O_2_ and releases NO under blue light (425 nm) irradiation, alongside controls **Ru‐16** and **Ru‐17**.

The Mao group were interested in combatting AMR by targeting biofilm formation. Zhao *et al*. therefore developed a ruthenium complex **Ru‐15** (Figure 17) which, upon light irradiation, can attack both bacteria and biofilms to treat resistant infections.^[57]^ The complex has two boronic acid groups which can selectively bind the surface of bacteria as well as biofilms. On irradiation the complex releases NO, which acts as a chemical signal to induce biofilm dispersal. The complex can also produce ROS to induce bacterial death. Control compounds **Ru‐16** and **Ru‐17** (Figure 17) were also synthesized without NO and without B(OH)_2_, respectively. Their NO and ^1^O_2_ generation abilities were assessed spectroscopically as well as by EPR and LC–MS. All three complexes generate ^1^O_2_ with similar *Φ*_Δ_ (∼0.2), while as expected only the NO containing complexes could achieve photorelease of NO. The complexes were assessed to determine their specificity of binding to bacteria over human cells.

*P. aeruginosa* (PAO1) and human fetal lung fibroblasts (WI‐38) were incubated with the three complexes and the zeta potentials determined. WI‐38 cells showed a small increase in zeta potential relative to the control with all three complexes (4–10 %). With PAO1 the complex without boronic acid groups, **Ru‐16** also induced an increase in zeta potential of 10 %. The two complexes with boronic acid groups (**Ru‐15** and **Ru‐16**) however, had an enhanced effect on PAO1 cells, with increased zeta potentials of 20–25 %. This result indicates enhanced interaction of the complexes with the bacteria due to the boronic acid groups. PAO1 and WI‐38 cells were also co‐incubated and the photocytotoxicity of the complexes assessed. In the dark, no change in viability was observed for either human or bacterial cells. Under blue light irradiation (425 nm, 20 mW cm^−2^, 20 min) all of the complexes displayed toxicity toward the bacteria and not towards the human cells. **Ru‐17** without boronic acid groups had the lowest activity with a 50 % reduction in viability, while **Ru‐16** and **Ru‐15** displayed 75 and 80 % reductions in PAO1 viability, respectively. The complexes biofilm inhibition properties were assessed alongside MB as a positive PDT control and diethylamine dinitric oxide (a NONOate) as a NO generation control. While all the complexes and MB displayed inhibitory activity, **Ru‐15** was by far the most potent with a 90 % reduction in bacterial viability and 80 % reduction in biofilm biomass. The complexes were further tested against three *P. aeruginosa* clinical isolates (PA22, PA36, and PA82) and similar results were obtained. The group further investigated the mechanism by which NO induces biofilm dispersal. Proteomic analysis showed that after exposure to **Ru‐15**, numerous proteins identified as key to quorum sensing and biofilm regulation were nitrosylated. This work demonstrates a powerful combination of bacterial selectivity, chemical signaling, and aPDT to eradicate PAO1 biofilms.

**Figure 18:**
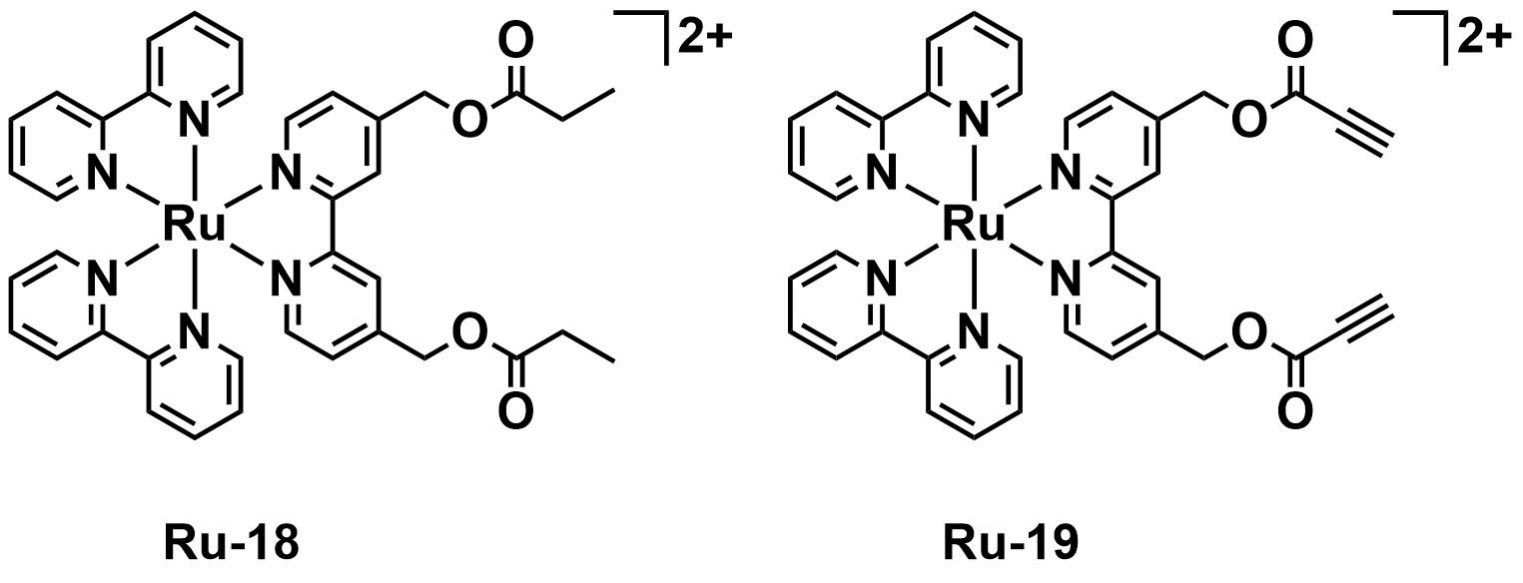
A ruthenium tris‐bipyridyl complex **Ru‐19** by Jian *et al*. capable of covalently binding to the surface of *S. aureus* and inducing cell death by ^1^O_2_ generation on exposure of blue light (470 nm) and control **Ru‐18**.

Jian *et al*. were also interested in targeting bacteria by binding to their surface. The group developed a [Ru(bpy)_3_]^2+^ complex with pendent alkyne moieties (**Ru‐19**, Figure 18), able to selectively and covalently bind to the surface of Gram‐positive bacteria on blue light irradiation (470 nm) in an amino‐yne click reaction.^[89]^ The complex can also generate ^1^O_2_ and thus induce bacterial cell death. A control without the alkyne groups **Ru‐18** (Figure 18) was also synthesized, and the two complexes were examined spectroscopically to determine their ^1^O_2_ *Φ*_Δ_. 9,10‐ADPA was used as a ^1^O_2_ trapping agent. Both complexes had *Φ*_Δ_ of ∼0.35. ^1^O_2_ generation was also confirmed by EPR spectrometry. The complexes were assessed for their photocytotoxicity against *S. aureus* (ATCC6538), MRSA (ATCC43300), and *E. coli* (ATCC25922). In general, the complexes displayed negligible dark toxicity and **Ru‐18** displayed no significant enhancement in activity on irradiation with blue light (470 nm, 22.5 mW cm^−2^, 20 min). **Ru‐19** was, however, found to be a potent photosensitizer with a log 5.52 reduction in CFUs (99.99 % inhibition) of MRSA on treatment at 10 μM with blue light (470 nm, 22.5 mW cm^−2^, 20 min). Activity against *E. coli* was expected to be reduced due to the lack of amines on the surface of Gram‐negative bacteria required for the amino‐yne click reaction. **Ru‐19** was however found to display a moderate effect with a log 3.34 reduction in CFUs under the same conditions. **Ru‐19** was tested against human normal ovarian epithelial cells (IOSE80) and L‐02 cells and found to display low dark toxicity with over 90 % viability for both cell lines with 10 μM **Ru‐19** and blue light irradiation (470 nm, 22.5 mW cm^−2^, 20 min).

Selective uptake of **Ru‐19** by *S. aureus* over IOSE80 and L‐02 was also demonstrated by CLSM. The complex was also found to cause very low levels of hemolysis in rabbit red blood cells even at 20 μM with blue light irradiation. This work further demonstrates the efficacy of targeting the bacterial surface to achieve selective toxicity in aPDT.

## 5 Conclusion and Outlook

In this review, we have covered the last five years of progress in the field of metal complexes for antimicrobial photodynamic therapy. The examples covered herein span a wide range of metals and ligands. The diversity of approaches is encouraging as it demonstrates the potential for growth in the field and the versatility of metal complexes for biological and optical applications. Recent advances demonstrate the range of possibilities available and how a PS's properties can be tuned by the correct choice of ligand, metal, and substituents.

As there is currently no standard or off‐the‐shelf PDT equipment, reported light sources and PDT assays vary a great deal. Equally the types of viability assay vary between the studies. Encouragingly, many do provide both MIC values and MBC values determined in a 96‐well‐plate concentration‐gradient assay and CFU counts allowing comparison between different papers. Some of the PSs achieve red‐light sensitization, which is advantageous for therapy deeper into tissue and biological media. ^1^O_2_ *Φ*_Δ_ is often determined to provide a rationale for the potency of a PS. This is a valuable technique; however, it does not always give the full story, particularly if the PS acts through type I PDT. In some cases, EPR was used to investigate the generation of ROS. This is the gold standard for radical species study and can provide invaluable insight into the mode of action of a PS, as each radical species gives a unique fingerprint. Another highlight is the number of clinical strains investigated. It is valuable to compare the results of PSs in both resistant and non‐resistant strains, for example, to demonstrate whether PDT can overcome current resistance mechanisms. In addition, some papers provided data on toxicity towards human cells, with examples achieving excellent selectivity towards bacteria. ICP‐MS and CLSM were used by several authors to demonstrate and quantify uptake. These techniques are powerful; however, further insights into the mechanisms of uptake and trends in uptake across series would be highly advantageous. In future, a more standardized approach to equipment, methods and reporting of data would be a huge asset to the field.

Some of the work reported herein has made excellent progress towards potent PSs that can address major challenges in aPDT, such as selective uptake by bacteria, red‐light activation, and antibiofilm properties. We hope this review helps others to better understand the challenges we face, inspires new and creative approaches to tackle AMR, and leads to high‐quality research in the future.

## Conflict of interest

The authors declare no conflict of interest.

6

## Biographical Information

*Thomas W. Rees obtained his PhD from the University of Birmingham (UK) and followed this with postdoctoral positions at Sun Yat‐Sen University (P. R. China) and the University of Nottingham (UK). He is now a Senior Laboratory Research Scientist at The Francis Crick Institute (UK). His research interests are in the synthesis of organometallic complexes for optoelectronic and biomedical applications. His current work focusses on metal complexes as novel classes of antimicrobials*.

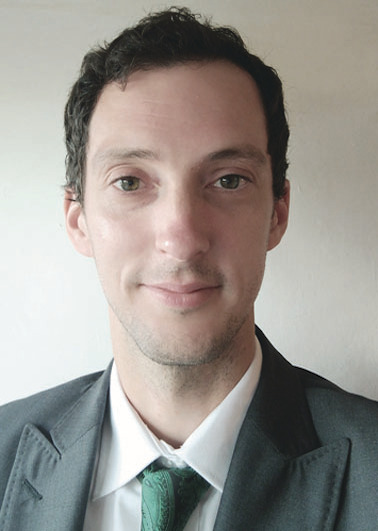

## Biographical Information

*Po‐Yu (Blake) Ho obtained his PhD in chemistry from Hong Kong Baptist University under the supervision of Prof Wai‐Yeung Wong. During this time, he visited the research group led by Prof. Richard Eisenberg at The University of Rochester (USA). After completing his PhD, he worked with Dr Sijie Chen at Karolinska Institutet (Hong Kong) as a postdoctoral researcher and changed his research direction from solar energy conversion materials to life science. He is currently working with Dr. Jeannine Hess at the Francis Crick Institute (UK) and King's College London (UK), and developing novel inhibitors and antibiotics based on metal complexes*.

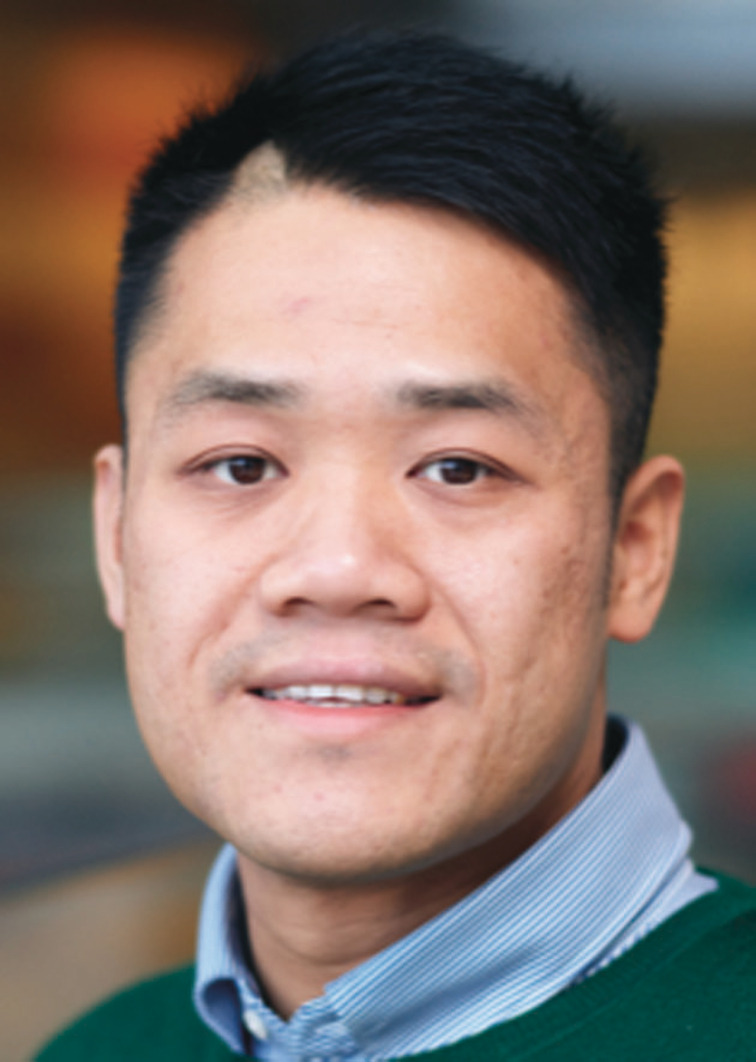

## Biographical Information

*Jeannine Hess obtained her PhD in chemical and molecular sciences in 2016 from the University of Zurich (Switzerland), where she developed metal‐based drug candidates as antiparasitic agents. She then joined the group of Prof. Chris Abell at the University of Cambridge (UK) with a SNSF Early Postdoc Mobility Fellowship and later a MSCA Individual Fellowship for her work on small‐molecule CoaBC inhibitors as new therapeutic modalities. In 2021, Jeannine was appointed as a Group Leader and Lecturer at the Francis Crick Institute (UK) and King's College London (UK). Her group's research focuses on rational and innovative approaches to develop metal‐based antimicrobials*.

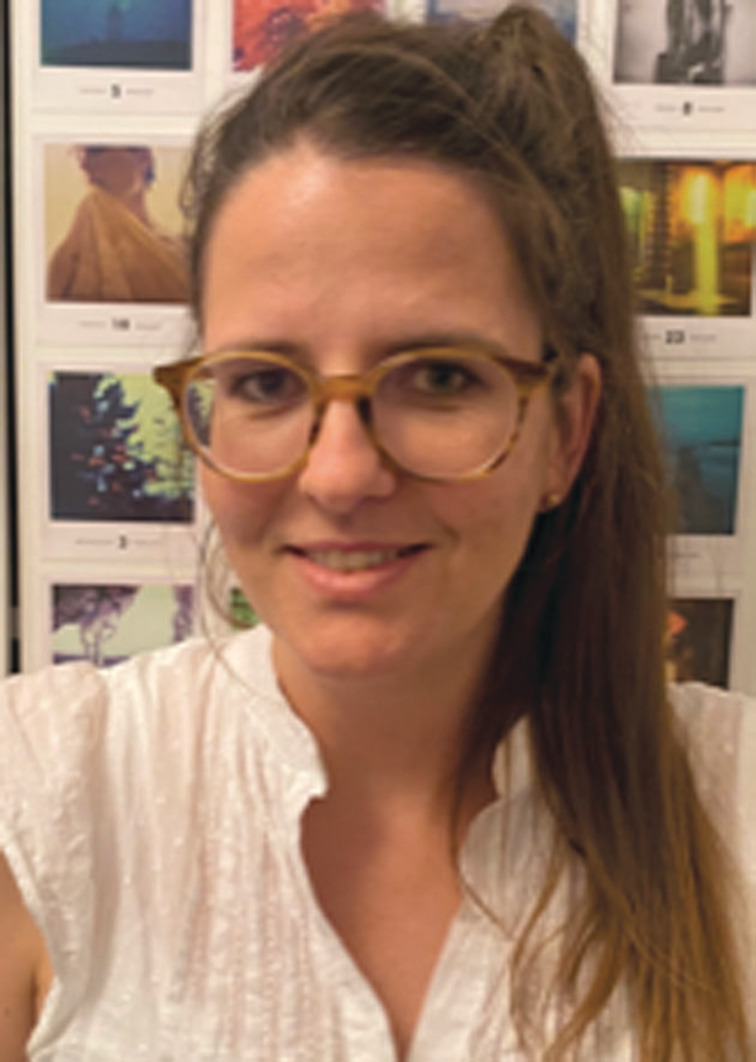

## Data Availability

Data sharing is not applicable to this article as no new data were created or analyzed in this study.
